# All-trans retinoic acid and interferon-α increase CD38 expression on adult T-cell leukemia cells and sensitize them to T cells bearing anti-CD38 chimeric antigen receptors

**DOI:** 10.1038/bcj.2016.30

**Published:** 2016-05-13

**Authors:** K Mihara, T Yoshida, S Ishida, Y Takei, A Kitanaka, K Shimoda, K Morishita, Y Takihara, T Ichinohe

**Affiliations:** 1 Department of Hematology and Oncology, Research Institute for Radiation Biology and Medicine, Hiroshima University, Hiroshima, Japan; 2 Department of Biochemistry, Nagoya University Graduate School of Medicine, Nagoya, Japan; 3 Division of Gastroenterology and Hematology, Department of Internal Medicine, Faculty of Medicine, Miyazaki University, Miyazaki, Japan; 4 Division of Tumor and Cellular Biochemistry, Department of Medical Sciences, Faculty of Medicine, Miyazaki University, Miyazaki, Japan; 5 Department of Stem Cell Biology, Research Institute for Radiation Biology and Medicine, Hiroshima University, Hiroshima, Japan

Survival of patients with adult T-cell leukemia (ATL), which is caused by human T-cell lymphotropic virus type-1 (HTLV-1), has been improved by the introduction of anti-CCR4 monoclonal antibody and allogeneic hematopoietic stem cell transplantation. However, not all patients benefit from these modalities, necessitating a novel therapeutic strategy.^1, 2^ Recently, an adoptive T-cell immunotherapy with chimeric antigen receptor (CAR) is clinically promising for patients with refractory blood diseases.^3, 4, 5, 6, 7, 8^ Thus, CD38 is an attractive target of CAR therapy for lymphoid neoplasms because it is widely expressed on cells of B- and T-lymphoid malignancies. We previously demonstrated marked cytotoxicity of T cells engineered to express anti-CD38-CAR against B-lymphoma cells and myeloma cells expressing CD38.^9, 10, 11^ To expand anti-CD38-CAR applicability against ATL cells that usually express undetectable or low CD38 levels, we must induce CD38 on the ATL cell surface. Interestingly, all-trans retinoic acid (ATRA), which is clinically used to treat patients with acute promyelocytic leukemia (APL), enhances CD38 expression on HL60 cells.^12^ Moreover, the upstream sequence of the CD38 gene contains an interferon regulatory factor-1 (IRF-1)-binding site. Here we show the marked cytotoxicity of anti-CD38-CAR T cells in HTLV-1-transformed cell lines as well as in cells from patients with ATL through the induction of CD38 expression by treatment with both ATRA and interferon (IFN)-α.

HTLV-1-transformed cell lines MT-2, MT-4, S1T, Su9T and ED were from Miyazaki University. Hut102 cells were obtained from the Cell Research Center for Biomedical Research (Sendai, Japan). Cells were cultured in RPMI-1640 medium containing 10% fetal calf serum and l-glutamine (Sigma, St Louis, MO, USA). ATL cells (acute type) from bone marrow, accounting for over 65% of mononuclear cells and peripheral blood, were provided after obtaining informed consent. These ATL cells and donors' cells were examined for approval by the institutional review board of Hiroshima University. A retroviral vector consisting of green fluorescent protein (GFP), CD8α, and 4-1BB, CD3ζ and anti-CD38 scFv was previously developed.^9, 10, 11^ Peripheral blood mononuclear cells were stimulated for 48 h with 7 μg/ml PHA-M (Sigma) and 200 IU/ml interleukin-2 (PeproTech, London, UK). T cells were transduced in an RD114-pseudotyped retrovirus-containing medium with 4 μg/ml polybrene (Sigma) in a retronectin-coated tube (Takara-Bio, Otsu, Japan) by spinoculation. An anti-CD38 antibody (CPK-H; MBL, Nagoya, Japan) was added to protect transduced T cells from autolysis through cross-linkage of anti-CD38-CAR with intrinsic CD38, as described previously.^10^ To detect anti-CD38-CAR, cells were stained with a goat anti-mouse (Fab')2 antibody-biotin (Jackson ImmunoResearch, West Grove, PA, USA), followed by PerCP–streptavidin (BD, Franklin Lakes, NJ, USA). Antibody staining was detected using a FACSCalibur flow cytometer (BD).^9, 10, 11^ For lactate dehydrogenase (LDH)-releasing cytotoxicity assay, cells (1 × 10^5^ cells per ml) were incubated with transduced T cells (1 × 10^5^ cells per ml) for 18 h at 37 °C in Opti-MEM medium (Invitrogen, Carlsbad, CA, USA). Solution containing tetrazolium salt and diaphorase was added to the supernatant collected before measuring absorbance using the LDH Cytotoxicity Detection Kit (Takara-Bio). To evaluate cytotoxicity of anti-CD38-CAR T cells, co-cultured cells were collected and stained with an anti-CD38 antibody-APC (BD). The specific cytotoxicity of anti-CD38-CAR T cells against CD38^+^ ATL cells treated with ATRA (Sigma) and/or IFN-α (PeproTech) was evaluated by flow cytometry.^9, 10, 11^

We first examined anti-CD38-CAR expression on retrovirally transduced human T cells from healthy donors using goat anti-mouse-IgG-PerCP, which cross-reacts with CAR. We confirmed that PerCP and GFP contained in the vector were co-expressed in transduced T cells (transduction efficiency: 61.26±10.66% (*n*=5)). Next, we investigated whether patients' ATL cells could be transduced with anti-CD38-CAR. GFP-positive T cells were negative for CD4 and CD25, indicating that ATL cells were not transduced with anti-CD38-CAR (Figure 1a). These results agreed with a previous observation that CD8^+^ T cells were markedly expanded and transduced with our methods.^10^ The transduction efficiency was 40.31±2.40% (*n*=4). Next, we evaluated the cytotoxicity of transduced T cells using the LDH releasing assay by co-incubating anti-CD38-CAR T cells with HTLV-1-transformed cell lines. As expected, MT-2 cells, with expression of CD38 being the highest among the six cell lines tested (97.06%), were efficiently abrogated by anti-CD38-CAR T cells (75.36±0.11% (*n*=3); Table 1). However, the other HTLV-I-transformed cell lines (MT-4, S1T, Hut102, Su9T and ED) lacking CD38 expression mostly survived after co-incubation with anti-CD38-CAR T cells (Table 1). Therefore, augmentation of CD38 expression was required to induce anti-CD38-CAR T-cell cytotoxicity against HTLV-1-transformed cell lines.

We investigated whether ATRA enhanced cytotoxicity of anti-CD38-CAR T cells by inducing CD38 expression on HTLV-1-transformed cell lines. As little as 10 nm of ATRA compared with an effective blood concentration for treating patients with APL, increased CD38 expression by over 80% in MT-4, S1T and Hut102 cells, but not in Su9T and ED cells (Figure 1b; Supplementary Figure 1a; Table 1). Three-day co-incubation of anti-CD38-CAR T cells with these cell lines at an effector (E): target (T) ratio of 1:2 in ATRA presence resulted in efficient elimination of MT-4, S1T and Hut102 cells according to the increased levels of CD38 expression (Figure 1b; Table 1). Cytotoxicity against cell lines was dependent on the number of T cells with anti-CD38-CAR in ATRA presence (Supplementary Figures 1b and c). Alternatively, ATRA withdrawal led to the basal level of CD38 expression of MT-4 cells before ATRA administration for 10 days (data not shown). However, CD38 induction by ATRA was insufficient to completely eliminate HTLV-1-transformed cells because 10–20% of S1T and Hut102 cells did not express CD38 in ATRA presence. To further improve the killing of HTLV-1-transformed cells and primary ATL cells by anti-CD38-CAR T cells through induced CD38 expression, we examined whether IFN-α and/or IFN-γ could enhance expression of the CD38 gene, whose upstream contains binding sites for IRF-1. IFN-α and IFN-γ efficiently enhanced CD38 expression in MT-4 cells even at a concentration below the therapeutic level, but not in Su9T, ED or S1T cells (Supplementary Figures 1a and c). As low as 2.5 U/ml of IFN-α induced CD38 expression in MT-4 cells for 18 h (CD38 expression: >95%). CD38 induction was more efficient with IFN-α than IFN-γ.

We then investigated whether ATL cells from patients were killed by anti-CD38-CAR T cells. Expression levels of CD38 in ATL cells from three patients varied (0.01–29.21%). Interestingly, 3-day treatment with 10 nm ATRA markedly enhanced CD38 expression in ATL cells from two of three patients (CD38 expression: 58.81 and 79.58%). Most importantly, anti-CD38-CAR T cells exerted marked cytotoxicity against ATL cells with CD38 expression enhanced by ATRA compared with T cells transduced with the vector control (Figure 1d; Table 1). However, CD38 induction by ATRA alone was much lower in patients' cells compared with that in cell lines. Thus, we examined whether the combination of ATRA with IFN-α enhanced surface CD38 expression. Notably, combined treatment with 10 nm ATRA and IFN-α synergistically enhanced CD38 expression in ATL cells from patients (CD38 expression: >90% Figure 1e; Table 1). ATRA and IFN-α did not reflect ATL cell numbers, because these reagents were used at extremely low concentrations (data not shown). Three-day co-culture of ATL cells from three patients with anti-CD38-CAR T cells in the presence of both ATRA and IFN-α at an E:T ratio of 1:2 resulted in eradication of >95% of ATL cells, demonstrating that they can be efficiently eliminated by anti-CD38-CAR T cells with both ATRA and IFN-α (Figure 1e; Table 1; Supplementary Figure 1d).

Treatment of ATL cells with both ATRA and IFN-α markedly enhanced the cytotoxicity of anti-CD38-CAR T cells against ATL cells through augmented CD38 expression. IFN-α partially suppressed ATL cell viability *in vitro*, suggesting an additional therapeutic benefit of IFN-α when used in combination with anti-CD38-CAR T cells.^13, 14^ CD38 is expressed in peripheral blood cells and restricted lineage-committed precursors in the bone marrow, as well as in the thymus and prostate. Thus, untoward toxicities in these organs may occur in anti-CD38-CAR T-cell therapy. Interestingly, an anti-CD38 antibody, daratumumab, has successfully been used to treat myeloma, indicating that therapeutic targeting of CD38 is a clinically feasible strategy. It has recently been reported that ATRA enhances the efficacy of daratumumab in myeloma patients whose myeloma cells expressed low levels of CD38 with fewer adverse effects.^15^ These findings suggest that ATRA may safely be used in combination with a CD38-targeting therapy. Further clinical studies are required to establish the safety and eligibility of ATL patients regarding the clinical use of anti-CD38-CAR T cells in combination with ATRA and/or IFN-α.

Patients receiving immunotherapy with anti-CD19-CAR T cells, which has a significant cytotoxicity against B-cell neoplasms, suffer from a prolonged B-cell aplasia and have to be periodically injected with γ-globulin. Furthermore, CAR therapy reportedly causes cytokine storm that can be lethal. Therefore, minimizing the cytotoxic activity of CAR T cells on normal cells, as well as augmenting expression of surface molecule on target cells, would be crucial in developing an effective therapy. Addition of a death domain to CAR may enable manipulation of CAR T cells to prevent the unwanted side effects before they occur. We envision that a novel immunotherapeutic strategy involving T cells carrying anti-CD38-CAR in combination with ATRA and IFN-α in the treatment of ATL may serve as a basis for the development of future CAR therapies.

## Figures and Tables

**Figure 1:**
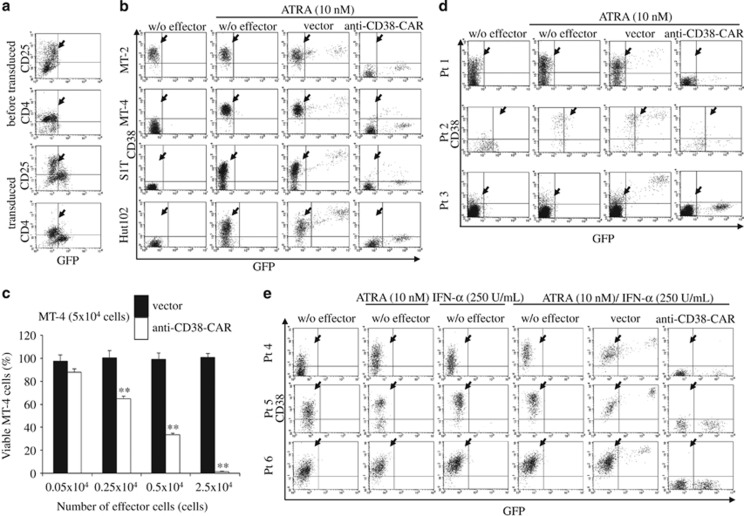
Cytotoxic effects of T cells expressing anti-CD38-CAR against HTLV-1-transformed cells and primary ATL cells in the presence of ATRA and/or IFN-α. (**a**) Peripheral blood cells from a patient with ATL cells were transduced. Cells were stained with anti-CD25 antibody-PE after transduction with the retroviral vector and then analyzed by flow cytometry. The ATL cell population is indicated by the arrowhead. (**b**) Four HTLV-I-transformed cell lines (MT-2, MT-4, S1T and Hut102 cells) were co-cultured with T cells transduced with vector alone or anti-CD38-CAR in the presence of 10 nm of ATRA at an E:T ratio of 1:2 for 3 days, respectively. The cells collected from the co-culture wells were stained with an anti-CD38 antibody-APC. The viable CD38^+^ cell population is indicated by the arrowhead. (**c**) MT-4 cells were co-incubated with T cells bearing an empty vector or anti-CD38-CAR vector in ATRA presence at various E:T ratios for 3 days. MT-4 cells were stained with an anti-CD38 antibody-APC followed by flow cytometry. Asterisks denote a significant difference between two adjacent columns. (**d**) ATL cells from three patients (Pt 1, Pt 2 and Pt 3) were co-cultured with T cells transduced with an empty vector or anti-CD38-CAR vector in the presence of ATRA at an E:T ratio of 1:2 for 3 days. Cells were collected and stained with anti-CD38 antibody-APC. The arrowhead indicates CD38^+^ cell populations. (**e**) ATL cells obtained from three other patients (Pt 4, Pt 5 and Pt 6) were co-cultured with T cells transduced with or without an empty vector or a vector carrying anti-CD38-CAR in the presence of ATRA, IFN-α or both at an E:T ratio of 1:2 for 3 days. Viable CD38^+^ cell populations are indicated by the arrowhead.

**Table 1:** Cytotoxicity of T cells expressing anti-CD38-CAR against HTLV-1-transformed cells and ATL cells

*Cells*	*Percentage of CD38*^*+*^ *cells (%)*	*Overall cytotoxicity of anti-CD38-CAR T cells by LDH assay (%)*	*Percentage of CD38*^*+*^ *cells with ATRA (%)*	*Specific cytotoxicity of anti-CD38-CAR T cells against CD38*^*+*^ *cells with ATRA by FCM (%)*	*Overall cytotoxicity of anti-CD38-CAR T cells with ATRA by FCM (%)*
MT-2	97.06±1.00	75.36±0.11	97.19±1.47	99.90±0.09	92.30±1.48
MT-4	2.91±0.31	4.97±1.18	97.81±0.36	98.61±0.12	97.92±0.33
S1T	0.01±0.01	0.73±0.34	81.34±1.35	96.98±0.09	81.08±1.12
Hut102	1.18±0.13	2.74±3.36	86.11±3.94	99.51±0.02	86.47±2.74
Su9T	0.05±0.04	1.61±1.49	0.04±0.03	ND	ND
ED	0.01±0.01	2.60±0.61	0.05±0.04	ND	ND
Patient 1	29.21±0.88	ND	58.81±1.24	99.84±0.22	58.70±1.11
Patient 2	5.24±0.89	ND	79.58±1.19	92.42±2.02	74.40±1.94
Patient 3	0.01±0.02	ND	0.04±0.01	ND	ND

*Cells*	*Expression of CD38 (%)*	*Expression of CD38 with ATRA (%)*	*Expression of CD38 with IFN-α (%)*	*Expression of CD38 with ATRA and IFN-α (%)*	*Overall cytotoxicity of anti-CD38-CAR T cells with ATRA/IFN-α by FCM (%)*
Patient 4	34.94±1.39	78.70±2.00	66.48±0.81	95.45±0.91	99.68±0.18
Patient 5	50.36±1.30	70.36±1.21	74.03±0.44	93.41±1.04	95.98±0.17
Patient 6	82.01±1.40	91.15±0.92	97.64±0.42	97.55±0.71	99.92±0.02

Abbreviations: ATL, adult T-cell leukemia; ATRA, all-trans retinoic acid; CAR, chimeric antigen receptor; FCM, flow cytometry; HTLV-1, human T-cell lymphotropic virus type-1; IFN-α, interferon-α LDH, lactate dehydrogenase; ND, not determined.

Results are the mean±s.d. for three experiments.

Specific cytotoxicity was evaluated by flow cytometry, following co-incubation of T cells bearing anti-CD38-CAR (E) with ATL cells (T) at an E:T ratio of 1:2 for 3 days.
