# Supplementary material for: All-trans retinoic acid and interferon-α increase CD38 expression on adult T-cell leukemia cells and sensitize them to T cells bearing anti-CD38 chimeric antigen receptors
Source: Blood Cancer J. 2016 May 13;6(5):e421–. doi: 10.1038/bcj.2016.30 (PMC4916299; doi:10.1038/bcj.2016.30)
Supplement: Supplementary Figure 1 [file bcj201630x1.doc]

**Supplementary Figure 1**

**Cytotoxiceffects of T cells expressing anti-CD38-CAR againstHTLV-1-transformed cells and primary ATL cells in the presence of ATRA and/or IFNs.**

**(a)**Su9T and ED cells treated with ATRA or IFN-αfor 3 days were stained with anti-CD38 antibody-APC and then subjected to flow cytometry. S1T cells were incubated with IFN-α or IFN-γ for 3 days. After being stained, cells were harvested and analyzed by flow cytometry.

**(b)**MT-4 cells were co-incubated without T cells in the upper panels, and co-cultured with T cells bearing vector alone as a control or anti-CD38-CAR vector in the presence of ATRA at various E: T ratios for 3 days, as indicated in the middle and lower panels. MT-4 cells were stained with anti-CD38 antibody-APC followed by flow cytometry.

**(c)**MT-4 cells were cultured with IFN-α or IFN-γ at various concentrations. CD38 expression was then evaluated by flow cytometry after staining with anti-CD38 antibody-APC.

**(d)**ATL cells from the patient were co-cultured with T cells transduced with an empty vector or anti-CD38-CAR in the presence of ATRA and/or IFN-α at an E: T ratio of 1: 2 for 3 days. The cells were harvested from the co-culture wells and stained with anti-CD38 antibody-APC and anti-CD25 antibody-PE. Thereafter, these cells were analyzed by flow cytometry.
