# Supplementary material for: All-trans retinoic acid and interferon-α increase CD38 expression on adult T-cell leukemia cells and sensitize them to T cells bearing anti-CD38 chimeric antigen receptors
Source: Blood Cancer J. 2016 May 13;6(5):e421–. doi: 10.1038/bcj.2016.30 (PMC4916299; doi:10.1038/bcj.2016.30)
Supplement: Supplementary Figure 1 [file bcj201630x2.ppt]

## Slide 1
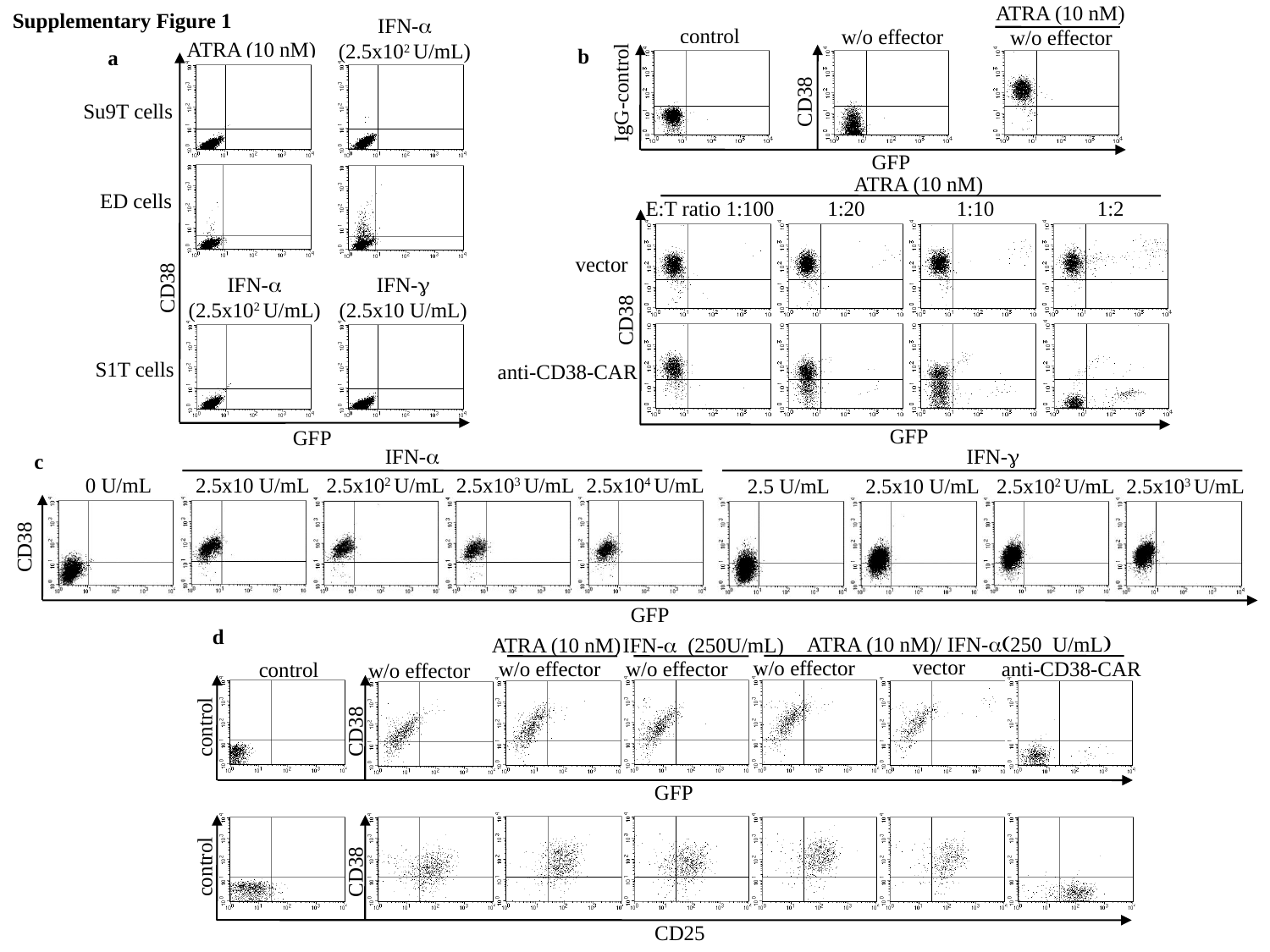

ATRA (10 nM)
Supplementary Figure 1
IFN-α
(2.5x10^2^ U/mL)
control
w/o effector
w/o effector
ATRA (10 nM)
b
a
IgG-control
CD38
Su9T cells
GFP
ATRA (10 nM)
ED cells
E:T ratio 1:100
1:20
1:10
1:2
vector
IFN-α
(2.5x10^2^ U/mL)
IFN-γ
(2.5x10 U/mL)
CD38
CD38
S1T cells
anti-CD38-CAR
GFP
GFP
IFN-α
IFN-γ
c
0 U/mL
2.5x10 U/mL
2.5x10^2^ U/mL
2.5x10^3^ U/mL
2.5x10^4^ U/mL
2.5 U/mL
2.5x10 U/mL
2.5x10^2^ U/mL
2.5x10^3^ U/mL
CD38
GFP
d
ATRA (10 nM)/ IFN- U/mL
ATRA (10 nM)
IFN-α (250U/mL)
vector
w/o effector
w/o effector
w/o effector
anti-CD38-CAR
control
w/o effector
control
CD38
GFP
control
CD38
CD25
